# Epidemiology, Public Health, and the Rhetoric of False Positives

**DOI:** 10.1289/ehp.0901194

**Published:** 2009-10-07

**Authors:** Aaron Blair, Rodolfo Saracci, Paolo Vineis, Pierluigi Cocco, Francesco Forastiere, Philippe Grandjean, Manolis Kogevinas, David Kriebel, Anthony McMichael, Neil Pearce, Miquel Porta, Jonathan Samet, Dale P. Sandler, Adele Seniori Costantini, Harri Vainio

**Affiliations:** 1 Division of Cancer Epidemiology and Genetics, National Cancer Institute, National Institutes of Health, Department of Health and Human Services, Bethesda, Maryland, USA; 2 IFC-National Research Council, Pisa, Italy; 3 Department of Epidemiology and Public Health, Imperial College London, London, United Kingdom; 4 ISI Foundation, Torino, Italy; 5 Dipartmento di Sanità Pubblica, Sezione de Medicina del Lavoro Asse Didattico–Policlinico Universitario, Monserrato (CA), Italy; 6 Department of Epidemiology, ALS, Roma, Italy; 7 Department of Environmental Medicine, University of Southern Denmark, 5000 Odense, Denmark; 8 Department of Environmental Health, Harvard School of Public Health, Boston, Massachussetts, USA; 9 Centre for Research in Environmental Epidemiology, Municipal Institute for Medical Research (IMIM), Bareclona, Spain; 10 CIBER Epidemiology y Salud Publica, Barcelona, Spain; 11 National School of Public Health, Athens, Greece; 12 Department of Work Environment, University of Massachusetts, Lowell, Massachussetts, USA; 13 National Centre for Epidemiology and Population Health, The Australian National University, Canberra, Australia; 14 Centre of Public Health Research, Massey University, Wellington, New Zealand; 15 Institut Municipal d’Investigació Mèdica and Universitat Autònoma de Barcelona, Catalonia, Spain; 16 Department of Preventive Medicine, Keck School of Medicine, University of Southern California, Los Angeles, California, USA; 17 Epidemiology Branch, Division of Intramural Research, National Institute of Environmental Health Sciences, National Institutes of Health, Department of Health and Human Services, Research Triangle Park, North Carolina, USA; 18 Unit of Occupational and Environmental Epidemiology, ISPO Cancer Prevention and Research Institute, Florence, Italy; 19 Finnish Institute of Occupational Health, Helsinki, Finland

**Keywords:** epidemiologic methods, false negatives, false positives, hyped findings

## Abstract

**Background:**

As an observational science, epidemiology is regarded by some researchers as inherently flawed and open to false results. In a recent paper, Boffetta et al. [Boffetta P, McLaughlin JK, LaVecchia C, Tarone RE, Lipworth L, Blot WJ. False-positive results in cancer epidemiology: a plea for epistemological modesty. J Natl Cancer Inst 100:988–995 (2008)] argued that “epidemiology is particularly prone to the generation of false-positive results.” They also said “the tendency to emphasize and over-interpret what appear to be new findings is commonplace, perhaps in part because of a belief that the findings provide information that may ultimately improve public health” and that “this tendency to hype new findings increases the likelihood of downplaying inconsistencies within the data or any lack of concordance with other sources of evidence.” The authors supported these serious charges against epidemiology and epidemiologists with few examples. Although we acknowledge that false positives do occur, we view the position of Boffetta and colleagues on false positives as unbalanced and potentially harmful to public health.

**Objective:**

We aim to provide a more balanced evaluation of epidemiology and its contribution to public health discourse.

**Discussion:**

Boffetta and colleagues ignore the fact that false negatives may arise from the very processes that they tout as generating false-positive results. We further disagree with their proposition that false-positive results from a single study will lead to faulty decision making in matters of public health importance. In practice, such public health evaluations are based on all the data available from all relevant disciplines and never to our knowledge on a single study.

**Conclusions:**

The lack of balance by Boffetta and colleagues in their evaluation of the impact of false-positive findings on epidemiology, the charge that “methodological vigilance is often absent” in epidemiologists’ interpretation of their own results, and the false characterization of how epidemiologic findings are used in societal decision making all undermine a major source of information regarding disease risks. We reaffirm the importance of epidemiologic evidence as a critical component of the foundation of public health protection.

Epidemiologic evidence has contributed substantially to science and to the protection and advancement of the public’s health through regulatory, legal, and medical pathways. Examples abound, including tobacco control motivated by the discovery that smoking causes a host of diseases, evidence-based regulation of environmental and occupational agents shown to cause cancer and other diseases, and identification of remediable risk factors for coronary heart disease. Scrutiny of the methods and results of epidemiologic research is often intense and sometimes adversarial given its critical contribution to public health, the vested interests involved (particularly for occupational and environmental causes of disease), and media attention. It is well known that observational epidemiologic studies may be affected by various biases that can impair their validity, and that are generally not present in experimental investigations. A strength of epidemiology is that it is based on real world conditions. Critical scrutiny of epidemiologic studies, covering all potential sources and mechanisms of biases, is indispensable. However, selective listing of evidence and an unbalanced discussion of possible methodologic flaws does a disservice to the discipline and to public health. For example, the charge of “false positive” results was misused for decades by the tobacco industry to discredit the overwhelming epidemiologic evidence linking active and passive smoking with cancer (United States of America et al. v. Philip Morris USA, Inc., et al. 2006). Similarly, reported health risks in modern life were ridiculed 20 years ago as mistaken menaces indicted by faulty “false positive” epidemiology (Feinstein 1988).

In a series of recent writings, Boffetta and coauthors (Boffetta et al. 2008, 2009a, 2009b) espouse the viewpoint that “false positive” results are a pervasive problem in epidemiologic research and, coupled with the tendency of epidemiologists to promote new findings, lead to decisions and actions that harm society. As a remedy Boffetta et al. argue strongly for a fundamental skepticism toward results from observational studies. This message could—and indeed is clearly intended to—have major scientific and public health implications. As epidemiologists involved for many years in the use, development, and teaching of epidemiologic methods, we believe this view not only is essentially flawed and overstated, but relegates observational studies to being little more than futile exercises to be regarded with skepticism and largely useless for decision making in public health. This conclusion would be a major blow to using epidemiology to advance public health.

## False Positives and False Negatives: Two Faces of One Coin

Consider the following hypothetical example: Suppose we repeatedly tossed a coin 100 times and recorded the number of “heads” and “tails.” About half of the time, the number of heads would be > 50% and there would be some instances when it was > 60%. Could we conclude on the basis of such an individual “case study” that found heads more frequent than tails that tossing a coin was biased toward producing more heads (false positives) than tails (false negatives)? Such a conclusion would be unjustified unless we had complete data. Collection of such data would likely show that the number of “false positives” (> 50 heads in 100 tosses) was about the same as the number of “false negatives,” (< 50 heads) and that the claim of an inherent methodologic bias was unjustified. Boffetta et al. (2008) discuss a few examples of what they consider false-positive findings and conclude that false-positive inferences are a major plague for epidemiology. Their deductive logic is flawed. It is ironic that they support their contention that false positives are a major problem with only a few anecdotal cases. If “false positives” were indeed such a widespread and serious problem in epidemiologic research, one would expect that it would be relatively easy to provide many more examples. From a few examples, the authors proceed to discuss seven general factors contributing to false-positive findings. We review and comment on these below.

### Multiple comparisons

Boffetta et al. (2008) offered developments in genome-wide association studies (GWAS) as support for their concern about multiple comparisons, but without acknowledging the basic difference between exploratory studies of thousands of genes with few prior hypotheses and studies focused on specific disease risk factors often with clear potential for adverse effects. Concern about multiple statistical testing comparisons is not merely a question of number of comparisons, but also how hypotheses and prior probability of associations are formulated (The Welcome Trust Case Control Consortium 2007). For example, in a study of dioxin and non-Hodgkin lymphoma, multiple comparisons would be of less concern because of a strong *a priori* hypothesis. Although statistical significance testing is used to address the potential role of chance in generating particular findings, estimates of effect from multiple studies typically drive decision making and societal action. Epidemiologic research on an issue is a process. Examples provided by Boffetta et al. (2008) of initial leads being unsupported by additional research demonstrate how the early phase of this process may be set aside after further investigation.

### Subgroup analyses

To reduce the risk of false-positive results, Boffetta et al. (2008) suggested that “the strategy for reporting study results should be specified before the results are known and selective reporting or emphasis of statistically significant results based on *ex post facto* subgroup analyses should be discouraged.” This is also a well-recognized concern. In practice, many scientific studies—epidemiologic and nonepidemiologic—have dual objectives of testing specifically stated hypotheses and estimating the size of the putative effect, while also conducting more exploratory analyses to provide leads for new relationships. Following their recommendation for *a priori* listing does not reflect current practice, which often involves exploration of already collected and readily available data. The need to follow up any new associations with more rigorous and often expensive studies is well recognized. A rigid application of their *a priori* specification would also be particularly restrictive and counter to the use of consortia and long-term prospective studies to evaluate risk factors or outcomes not specified at the design stage of the studies. Some of the authors (Boffetta et al. 2008) are themselves engaged in prospective studies and pooling projects and their recommendation belies their own practice.

### Information bias and selection bias

All standard courses in epidemiologic methods caution that these biases may lead to over- or underestimation of risk. Boffetta et al. (2008) mentioned only overestimation. In particular, it is well established that if misclassification of exposure (or disease) is “nondifferential”—that it is unrelated to disease status (or exposure status)—then the resulting bias will typically be toward the null value. Such information bias will tend to diminish the size of any observed association and is more likely to lead to a conclusion of no association than to a false-positive one. Because nondifferential exposure misclassification is a common occurrence, even in well-designed epidemiologic studies, its consequence would be quite opposite of false-positive concerns. On balance, epidemiologic research that involves misclassified exposure data has an inherent bias toward false-negative rather than false-positive findings, contrary to the arguments of Boffetta et al. (2008).

### Confounding

Boffetta et al. (2008) cited a statistical simulation showing that uncontrolled confounding can account not only for relative risks just above 1, but also for risks in the 1.5–2.0 range (Fewell et al. 2007). Epidemiologists are well aware of the potential for confounding to introduce noncausal associations and generally take steps in the design and analysis phases of research to address confounding. Simulations mentioned by the authors offer a reminder of the possible consequences of residual confounding. We agree that there is a potential for uncontrolled confounding; however, its frequency is critical to understanding the magnitude of the implied problem. Among relevant evidence, studies of occupation and lung cancer have found smoking to be a relatively infrequent confounder despite the magnitude of the association between smoking and lung cancer risk. In this specific example, the relative risk of the disease of interest (e.g., lung cancer) from the putative confounder (i.e., smoking) is very large and smoking prevalence is known to vary by occupation. Yet confounding due to smoking of occupational lung cancer risks is in practice a rare occurrence (Blair et al. 1985, 2007; Siemiatycki et al. 1988; Simonato et al. 1988). Finally, confounding can both increase and decrease relative risks, although the latter possibility is overlooked by Boffetta et al. (2008).

### Overestimation of effects in initial and/or small randomized trials

Small (“under-powered”) randomized trials sometimes generate statistically significant but false-positive results that are later refuted by subsequent larger trials. The same may occur in observational studies. Most major randomized trials today, however, are adequately powered. These points are not new, and no evidence is provided to indicate that the problem of underpowered studies is unappreciated, that it is more severe than in other scientific disciplines, or that it leads to mistaken decisions in public health. Unmentioned is the reverse problem—a false-negative finding which can also occur from an underpowered trial.

### Publication bias

As evidence that negative studies go unpublished, Boffetta et al. (2008) provided one example, dioxin exposure and risk of non-Hodgkin’s lymphoma, that shows a deficit of small negative studies and an excess of small positive ones. There are reasons to believe that publication bias may sometimes occur, but its frequency is unknown. We would note that today researchers are highly motivated to publish even negative results because of the expense of conducting studies and the intense public debate that surrounds public health issues.

## Factors Producing False Negatives

Although Boffetta et al. (2008) concluded that much epidemiologic research on cancer is at risk for false-positive findings and incorrect causal inference, they ignored mechanisms that would tend to produce false-negative results. In fact, false negatives can be produced by many of the same factors that they cited as causing false-positive findings, as well as by nondifferential misclassification of exposure and disease and use of crude measures of association. Nondifferential misclassification, unlike other mechanisms of bias, is probably always present to some degree in epidemiologic studies. Because it tends, on average, to reduce the observable risk below the true value in cohort and case–control studies, that is, to induce false-negative results, this effect can be substantial. For example, if 20% of workers are exposed to an occupational hazard that produces a real increase in the risk of lung cancer of 50%, a nondifferential misclassification arising from an exposure estimation method with sensitivity and specificity both equal to 80% will reduce the observable increase in lung cancer risk to about 20%. A relative risk of 1.2 is in the range where it might be difficult to conclude that there is an association. Thus, reduction in calculated relative risk can be sizable even when sensitivity and specificity are as high as 80%. The reduction is likely to be greater in many epidemiologic studies evaluating occupational or environmental exposures, because few available exposure classification methods have such a high sensitivity and specificity. When more than two levels of exposure are evaluated, nondifferential misclassification can result in an increase in relative risks for exposure categories other than the highest (Dosemeci et al. 1990). This effect, however, would tend to confuse an exposure–response gradient and diminish confidence that a true positive association exists. In other words, it would tend to lead to false-negative conclusions.

Crude assessments of exposure can also lead to underestimation of risk and to false-negative conclusions. For example, the relative risk of lung cancer in the classic British doctors study ranges from 5.6 for regular smokers of 1–4 cigarettes per day to 50.7 for the smokers of 35–40 cigarettes (Doll and Peto 1978). A simple binary categorization of persons as either smokers or nonsmokers, however, produces a relative risk of 12.0, which greatly underestimates the risk at higher levels of exposure (Doll and Peto 1978). In occupational epidemiology, classification of exposure as ever versus never and duration have often been the only available proxies of the actual exposures and are typically cruder and weaker exposure scales than cumulative or intensity-weighted metrics. Imprecise exposure assessment tends to reduce relative risks. For example, in the classic study by Redmond et al. (1972), the standardized mortality ratio (SMR) for lung cancer among workers who ever worked in the coke plant at a steel mill was 1.7. But this risk was almost entirely explained by the risk to a small group of workers whose duties kept them on top of the coke ovens and for whom the SMR was 10.0 for those with ≥ 5 years on the job.

## Hyped Findings

Boffetta et al. (2008) claimed that damage caused by false-positive results is further enhanced because they are “hyped” by epidemiologists and the press. We recognize that the media may over interpret study findings, regardless of the field of research. Of course, legitimate differences in interpretation of study results may occur among epidemiologists. Boffetta et al. (2008) provided no indication as to when differences in interpretation between scientists should be classified as “hyped” and when they are legitimate disagreements. They implied that such “hyping” is more common with epidemiology than with other scientific disciplines, but the evidence they provide to support the claim is entirely anecdotal. Furthermore, when economic interests are at stake, there is ample opportunity for the alternative views to be heard through press releases from affected groups used by the media and the common tendency for reporters to seek comments from individuals and groups with different points of view.

For the papers labeled as “hyped” by Boffetta et al. (2008), we reviewed how the authors of these papers summarized their findings.

### Dichlorodiphenyltrichloroethane (DDT) and breast cancer

“These findings suggest that environmental chemical contamination with organochlorine residues may be an important etiologic factor in breast cancer. Given the widespread dissemination of organochlorine insecticides in the environment and the food chain, the implications are far-reaching for public health intervention worldwide” (Wolff et al. 1993).

### Accompanying editorial on the DDT breast cancer paper

“These data [Wolff et al. 1993], although limited, do suggest the plausibility of an association between organochlorines and increased risk of breast cancer. However, at this stage, these mechanisms are incompletely understood, and they will require considerable additional refinement before becoming truly compelling” (Hunter and Kelsey 1993). Thus, the accompanying editorial placed these results in the appropriate context.

### Induced abortion and breast cancer

“Our data support the hypothesis that an induced abortion can adversely influence a woman’s subsequent risk of breast cancer. However, the results across all epidemiologic studies on this premise are inconsistent—both overall and within specific subgroups” (Daling et al. 1994).

### Pancreatic cancer and coffee

“This association should be evaluated with other data; if it reflects a causal relation between coffee drinking and pancreatic cancer, coffee use might account for a substantial proportion of the cases of this disease in the United States” (MacMahon et al. 1981).

These summary sentences and the implications highlighted by the authors vary, but none of them claimed that their results provide sufficient evidence to conclude that the association is causal, nor do they call for public action. Although the findings of each study had potential implications for public health, additional publications quickly led to a judgment that a causal association was unlikely, thereby confirming the importance of replication and consistency in inferring causality and the value of epidemiologic research in generating the evidence base for public health protection.

We do not find obvious evidence of hype in the papers so labeled by Boffetta et al. (2008). The article with the strongest statement, on breast cancer and DDT, was accompanied by an editorial that indicated considerable additional information was required before the hypothesis could be regarded as “truly compelling.”

Although we disagree with Boffetta et al. (2008) regarding “hype” in the above-mentioned articles, this may reflect a genuine difference in judgment in regard to the strength of evidence necessary to make any positive statement about a possible association. To evaluate this point, we selected papers by authors of the Boffetta et al. (2008) commentary on topics with relatively few previous publications and lack of consensus on causation to assess how these authors describe conclusions from their own data.

### Antidepressants and non-Hodgkin lymphoma

“Our results indicate an increased risk of non-Hodgkin’s lymphoma specifically among long-term users of tricyclic antidepressant medications” (Dalton et al. 2008). Two previous papers on the topic were cited in this paper; one was positive and one was negative.

### Flavonoids and laryngeal cancer

“This study provides support for a beneficial effect of selected flavonoids on laryngeal cancer risk” (Garavello et al. 2007). The authors cited one previous case–control study of 34 laryngeal cancer cases, although papers were available linking flavonoids with other cancers.

### Formaldehyde and laryngeal cancer

“A possible link between high formaldehyde exposure and laryngeal cancer was suggested” (Shangina et al. 2006). There were no previous studies supporting a link between formaldehyde and laryngeal cancer.

### Acrylonitrile and lung cancer

“Exposure to acrylonitrile was associated in our study with risk of lung cancer” (Scelo et al. 2004). This association between acrylonitrile and lung cancer reported in another paper was specifically presented by Boffetta et al. (2008) as an example of a hyped false positive.

We find the interpretative language used in previous papers by authors of the Boffetta et al. (2008) paper to be no different from those cited as examples of “hype.”

In conclusion, the claim by Boffetta et al. (2008) for a widespread problem of “hyping” is based on anecdotal evidence. In epidemiologic terms, they use a few “case reports” (and we see little evidence of “hyped” interpretations in these) to draw a causal connection between false positives and hyping. Even if this so-called hyping does occur on occasion, it is not so widespread as to justify the serious charge that epidemiologists do not appropriately evaluate their data and that they actively seek to publicize unsubstantiated findings in only one direction. Although similar accusations were made 20 years ago (Feinstein 1988), our accumulated experience today is just the opposite. We find that epidemiologists focus intensively on possible sources of bias in individual studies and in their review of the evidence.

## Epidemiology: Futile or Essential to Public Health Decisions?

The argument by Boffetta et al. (2008) on the negative impact of false positives on public health also rests on a faulty characterization of the process of achieving scientific consensus on public health issues. They implied that a single false-positive study leads directly to some societal action. Only under extraordinary circumstances are actions taken on the basis of a single study. In our considerable personal experience, we find that scientists serving on evaluative groups, such as IARC (International Agency for Research on Cancer) Monograph Working Groups, carefully consider data from all relevant scientific disciplines and pay special attention to the strengths and weaknesses of the many studies being considered.

Boffetta et al. (2008) were concerned about false-positive findings because they “may lead to inappropriate government and public health decisions, including the introduction of costly and potentially harmful measures.” Public health actions based on invalid data would be a concern. Resources are always limited and useless actions might preclude more beneficial activities from being taken elsewhere, but they provided no clear examples where this has occurred. They also focused only on false-positive results and ignore the consequences of false-negative findings, which may also have deleterious societal impacts. The false appearance of a lack of an increase in risk, or of only a small increase in risk, may result in inaction when action is, in fact, warranted. Failure to act may result in avoidable mortality and morbidity, which has human and economic costs (e.g., in medical care and lack of economic productivity). Another serious consequence that is rarely addressed is that false-negative findings may not motivate replication. In contrast, a new positive finding tends to generate scientific interest and activity, and the true relationship will ultimately be elucidated. This was aptly demonstrated in the previously mentioned association reported between coffee and pancreatic cancer (MacMahon et al. 1981), which was quickly evaluated in several other studies and, in fact, not confirmed.

## Conclusions

We find that the commentary by Boffetta et al. (2008) offers an unbalanced view of false-positive results in epidemiologic research and an overinterpretation of their consequences for public health. We have documented the lack of evidence in support of their arguments. Their commentary rhetorically attacks a “straw man” that the authors have themselves erected based on a few selected examples, which are extrapolated to the conclusion that “false-positive results are a common problem” and that “users of epidemiological results outside the scientific community … should be aware of the fact that statistically significant or positive results are often false.”

This dismissal of epidemiology fails on two crucial points with regard to its actual practice. First, epidemiologic evidence is usually considered in a context of relevant findings from other scientific disciplines during evaluations of topics of public health significance. Thus, a careful interpretation of the results from any epidemiologic investigation calls for examination of the findings in the light of all available scientific evidence. In fact, decisions made by regulatory agencies and public health–oriented institutions like IARC are based on minimizing the impact of both false-positive and false-negative results and on a comprehensive evaluation of all relevant scientific data, not just epidemiology (IARC 2008). Moreover, conclusions of the IARC Monographs establishing human carcinogenicity of an exposure have never been based, to our knowledge, on a single study. Thus, the concern by Boffetta et al. (2008) that false-positive findings from an epidemiologic study would lead to “inappropriate governmental and public health decisions” postulates a scenario that seems unlikely and certainly was not documented by any examples offered.

Second, decisions for societal action, whether in clinical medicine, public health, or politics, must necessarily be based on the totality of evidence available at the time of decision. In an exchange of letters subsequent to publication of their 2008 paper, Boffetta et al. (2009a) stated that

committee reports and their conclusions in themselves should not be misconstrued as science: they are consensus documents and opinions with an eye towards closure. In contrast, science is inherently open-ended, provisional in its findings and conclusions.

This is correct, but decisions must be based on the evidence currently in hand. As stated by Hill (1965),

in asking for very strong evidence I would, however, repeat emphatically that this does not imply crossing every ‘t’, and swords with every critic, before we act. … All scientific work is incomplete—whether it be observational or experimental. All scientific work is liable to be upset or modified by advancing knowledge. That does not confer upon us a freedom to ignore the knowledge we already have, or to postpone the action that it appears to demand at a given time.

Skepticism toward epidemiologic results and open-ended waiting for action until a final truth emerges that is satisfactory to all segments of society will guarantee only that important actions are delayed, as the history of the successful efforts of the tobacco industry to retard anti-smoking actions clearly demonstrates. Although science should heed proper skepticism, it should not do so to such an extent that new ideas are stifled. It is of interest that in the alleged spirit of epistemologic modesty, such slogans as “sound science” and “evidence-based toxicology” have been put forward by professionals with a record of collaboration with the tobacco industry (Guzelian et al. 2005). The purpose has clearly been to dismiss evidence from animal models and only accept “conclusive” epidemiology on risks in humans (Ruden and Hansson 2008), effectively cutting off a line of important evidence for risk assessment. Epidemiology is an important research component of public health. Boffetta et al. (2008) appear to relegate it to the role of a futile exercise producing scientific papers of doubtful utility, whose results should be regarded with such skepticism that they can be comfortably exempted from policy and practice implications and decisions. We reject this contention on scientific grounds and believe that it would be damaging to public health and to society at large. It is the responsibility of epidemiologists to design and conduct studies in a way that makes them capable of assisting public health and clinical decisions. We also believe that an evaluation of epidemiologic findings based on a balanced weighing of potentials for false-positive and false-negative biases along with other considerations of strengths and weaknesses within the framework of all other pertinent scientific evidence can and does produce valid scientific knowledge essential to public health actions and to advancement of science.
